# Probiotic *Lactobacillus acidophilus* Strain INMIA 9602 Er 317/402 Administration Reduces the Numbers of *Candida albicans* and Abundance of Enterobacteria in the Gut Microbiota of Familial Mediterranean Fever Patients

**DOI:** 10.3389/fimmu.2018.01426

**Published:** 2018-06-26

**Authors:** Astghik Pepoyan, Marine Balayan, Anahit Manvelyan, Lilit Galstyan, Sofi Pepoyan, Susanna Petrosyan, Vardan Tsaturyan, Shigeru Kamiya, Tamas Torok, Michael Chikindas

**Affiliations:** ^1^Department of Food Safety and Biotechnology, Armenian National Agrarian University, Yerevan, Armenia; ^2^International Association for Human and Animals Health Improvement, Yerevan, Armenia; ^3^Yerevan State Medical University, Yerevan, Armenia; ^4^Armenian National Agrarian University, Yerevan, Armenia; ^5^Kyorin University School of Medicine, Tokyo, Japan; ^6^Lawrence Berkeley National Laboratory, Berkeley, CA, United States; ^7^Health Promoting Naturals Laboratory, School of Environmental and Biological Sciences, Rutgers State University, New Brunswick, NJ, United States

**Keywords:** familial Mediterranean fever, M694V/V726A mutations, gut microbiota, gender, *Candida albicans*, *Lactobacillus acidophilus* INMIA 9602 Er 317/402, *Enteroccocus faecalis*, *Enterobacteriaceae*

## Abstract

Intestinal microorganisms play a crucial role in health and disease. The disruption of host–microbiota homeostasis has been reported to occur not only during disease development but also as a result of medication. Familial Mediterranean fever (FMF) is an inflammatory genetic disease characterized by elevated systemic reactivity against the commensal gut microbiota and high levels of *Candida albicans* in the gut. This study’s major objective was to investigate the effects of commercial probiotic Narine on the relative abundance of gut bacteria (specifically, enterobacteria, lactobacilli, *Staphylococcus aureus*, and enterococci) of *C. albicans* carrier and non-carrier FMF patients in remission. Our main finding indicates that the probiotic reduces numbers of *C. albicans* and abundance of enterobacteria in male and female patients of *C. albicans* carriers and non-carriers. It has pivotal effect on *Enterococcus faecalis*: increase in male non-carriers and decrease in female ones regardless of *C. albicans* status. No effect was seen for *Lactobacillus* and *S. aureus*. Our data suggest that M694V/V726A pyrin inflammasome mutations leading to FMF disease may contribute to gender-specific differences in microbial community structure in FMF patients. The study’s secondary objective was to elucidate the gender-specific differences in the gut’s microbial community of FMF patients. The tendency was detected for higher counts of enterobacteria in female FMF subjects. However, the small number of patients of these groups preclude from conclusive statements, pointing at the need for additional investigations with appropriate for statistical analysis groups of subjects involved in the study.

## Introduction

*Candida albicans* is an opportunistic pathogen, which often exists as a harmless human commensal microorganism (1). Mutually beneficial associations of *C. albicans* were reported with several members of the intestinal microbiota (2, 3). In contrast, cooperative interaction of *C. albicans* and *Escherichia coli* increased the probability of fungal urinary tract infections due to increased *E. coli*-induced adhesion of *C. albicans* to the bladder mucosa (4). Another possibly synergistic interaction was shown between *Staphylococcus aureus* and *C. albicans* (5).

The disruption of inflammasomes, intracellular protein complexes with an important role in the sensing of intracellular pathogen- and danger-associated molecular patterns, can lead to susceptibility to infection, gut auto-inflammation, and tumorigenesis (6–8). Disruption of the host immune system—intestinal microbiota’s homeostasis is the main cause of inflammatory bowel disease. In particular, *Klebsiella pneumoniae* and *P. mirabilis* were found to be associated with colitis in animals using the T-bet^−/−^ × Rag2^−/−^ ulcerative colitis model (9).

Separate from this is the gene responsible for another inflammatory disease: familial Mediterranean fever (FMF), designated *MEFV*, which encodes an inflammasome pyrin containing domain of purin (PYD), TRIM, and B30.2 and is activated by bacterial toxins like *Clostridium difficile*, toxin B (TcdB), and C3 toxins (10). *MEFV*, in addition to FMF, can cause pyrin-associated auto-inflammation with neutrophilic dermatosis (PAAND) (11). The PYD is detected in more than 20 human proteins related to inflammation and apoptosis, such as NLRP_3_ (12). A methionine residue at position 694, important for conservation of the pyrin’s structure, is associated with a severe form of FMF (prevalent among Armenian patients). Rowczenio and co-authors assumed that the location of p.M694 in the putative binding site of caspase-1 (the substitution/deletion of methionine) may promote IL-1β generation by inhibition of pyrin–caspase-1 interactions (13). The B30.2/SPRY domain may also interact with viral components (14).

Elevated systemic reactivity against commensal gut microbiota has been reported in Armenian FMF patients. In many patients, inflammation can persist during an attack-free period. It has been revealed that IgG antibodies against *Bacteroides, Parabacteroides, Escherichia*, and *Enteroccocus* antigens are significantly increased (15). It was also shown earlier that the presence of *C. albicans* above 10^3^ CFU/g of fecal materials is a common problem for Armenian FMF patients. While no participant had used antibiotics in the 2–3 months prior to the investigation, 45.5% of patients carried *C. albicans* in relatively high numbers (more than 10^3^ CFU/g fecal materials) (16). Culturing revealed high numbers of the bacterial genera *Proteus, Klebsiella, Enterobacter*, and *Citrobacter* at cell densities of more than 10^3^ CFU/g fecal materials in FMF patients (17). Probiotic strains may have different clinical effects, and the efficacy of one strain does not indicate that the other strains will be similarly efficacious as described (18).

The probiotic *Lactobacillus acidophilus* INMIA 9602 Er strain 317/402 was isolated in 1963 from the gut microbiota of a healthy newborn infant and named Narine after the investigator’s daughter (19). Studies have revealed that the strain has antagonistic activities against a wide range of pathogenic Gram-positive and Gram-negative microorganisms, including several clinical isolates of *Clostridium difficile* (20), *Bacillus subtilis, Pseudomonas aeruginosae*, and *Klebsiella pneumonia* (21), and *Cronobacter sakazakii* (22). The normalization of erythrocyte sedimentation rate (ESR) and levels of C-reactive protein (CRP) in patients’ blood after consumption of a Narine probiotic formulation (Vitamax-E, Armenia) was observed in a double blind, partially randomized, placebo-controlled trial of 30 volunteer patients with FMF in remission (23). Moreover, regulatory action of this strain on gut commensal *E. coli* was shown in FMF patients (24). *L. acidophilus* INMIA 9602 Er 317/402 had no *in vitro* impact on *C. albicans* strains isolated from FMF patients (16).

This study examined the effect of *L. acidophilus* INMIA 9602 Er 317/402, isolated from the commercial probiotic formulation used by Armenian FMF patients, on the relative abundance of gut enteric bacteria, lactobacilli, *S. aureus*, and *E. faecalis* in *C. albicans*-carrier FMF patients with the MEFV pyrin inflammasome mutation M694V/V726A, which is the prevalent pattern in the Armenian cohort. Main research questions were to show (i) if the changes in gut microbiota of FMF patients, primarily associated with the M694V/V726A pyrin inflammasome mutations, could lead to overgrowth of gut *C. albicans* of the patients and (ii) if colchicine/probiotic could effect on gut microbiota of patients through the regulation of NLRP inflammasomes.

## Materials and Methods

Forty healthy volunteers (20 male and 20 female) with less than baseline levels of *C. albicans* in the gut microbiota and without mutations in *MEFV* and 48 FMF volunteers (24 males and 24 females) were enrolled in a double-blind, partly randomized, placebo-controlled trial. Out of these participants, only 30 healthy (8 males and 22 females) and 31 (20 males and 11 females; 15 *C. albicans* non-carriers and 16 *C. albicans* carriers, see the Table 1) completed the trial. The probiotic *Lactobacillus acidophilus* INMIA 9602 Er 317/402 strain (Narine, Vitamax-E, Armenia) were prescribed to patients in each Narine FMF group. Remaining patients took placebo (capsule without the probiotic). The study participants took one capsule of placebo or probiotic preparation (150 mg of the probiotic strain contained no less than 1.5 × 10^8^ of viable bacteria) twice a day for 30 days. The datasets generated for this study can be found here: https://www.ncbi.nlm.nih.gov/geo/info/linking.html (GSE111835 study at: https://www.ncbi.nlm.nih.gov/geo/query/acc.cgi?acc=GSE111835). Besides, another study on the effect of Narine probiotic on gut *C. albicans* of 12 FMF patients with *C. albicans* counts above 10^6^ CFU/g fecal material (7 males and 5 females) were completed during these investigations.

**Table 1:** Hybidization scores for gut bacteria in *Candida albicans*-carrier and non-carrier familial Mediterranean fever (FMF) patients after probiotica^a^ therapy; average ± SD.

Bacteria	Control FMF group (*N* = 31)				Placebo group^b^ (*N* = 15)				Narine group^c^ (*N* = 16)			
	Male (*N* = 20)		Female (*N* = 11)		Male (*N* = 9)		Female (*N* = 6)		Male (*N* = 9)		Female (*N* = 7)	
	*C. albicans* − *N* **=** 12	*C. albicans* + *N* = 8	*C. albicans* − *N* = 3	*C. albicans* + *N* = 8	*C. albicans* − *N* = 6	*C. albicans* + *N* = 3	*C. albicans* − *N* = 3	*C. albicans* + *N* = 3	*C. albicans* − *N* = 4	*C. albicans* + *N* = 5	*C. albicans* − *N* = 4	*C. albicans* + *N* = 3
*Enterobacteriaceae* spp.	5,208 ± 459	4,853 ± 378	6,417 ± 347	8,141 ± 473	6,992 ± 558	5,267 ± 325	9,590 ± 794	9,780 ± 422	5,046 ± 578	4,483 ± 429	4,062 ± 529	6,883 ± 789
		*P*_1_ > 0.05		*P*_1_ < 0.05	*P*_2_ < 0.05	*P*_2_ > 0.05	*P*_2_ < 0.05	*P*_2_ < 0.05	*P*_2_ > 0.05	*P*_1_ > 0.05		*P*_1_ > 0.05
									*P*_3_ < 0.05	*P*_2_ > 0.05	*P*_2_ < 0.05	*P*_2_ > 0.05
										*P*_3_ < 0.05	*P*_3_ < 0.05	*P*_3_ < 0.05

*Lactobacillus* spp.	3,556 ± 97	3,192 ± 107	4,112 ± 93	2,834 ± 112	4,560 ± 299	4,360 ± 273	4,978 ± 138	3,813 ± 201	4,793 ± 289	3,834 ± 263	4,873 ± 185	3,941 ± 217
		*P*_1_ < 0.05		*P*_1_ < 0.05	*P*_2_ < 0.05	*P*_2_ < 0.05	*P*_2_ < 0.05	*P*_2_ < 0.05	*P*_2_ < 0.05	*P*_2_ < 0.05	*P*_2_ < 0.05	*P*_2_ < 0.05
									*P*_3_ > 0.05	*P*_3_ > 0.05	*P*_3_ > 0.05	*P*_3_ > 0.05

*Enterococcus faecalis*	4,441 ± 128	4,928 ± 144	6,405 ± 157	4,168 ± 213	6,374 ± 169	5,689 ± 109	6,534 ± 205	5,070 ± 244	7,108 ± 273	5,418 ± 211	5,602 ± 189	3,771 ± 123
		*P*_1_ < 0.05		*P*_1_ < 0.05		*P*_1_ < 0.05		*P*_1_ < 0.05		*P*_1_ < 0.05		*P*_1_ < 0.05
					*P*_2_ < 0.05	*P*_2_ < 0.05	*P*_2_ < 0.05	*P*_2_ < 0.05	*P*_2_ < 0.05	*P*_2_ > 0.05	*P*_2_ < 0.05	*P*_2_ < 0.05
									*P*_3_ < 0.05	*P*_3_ > 0.05	*P*_3_ < 0.05	*P*_3_ < 0.05

*^a^*Lactobaillus acidophilus* INMIA 9,602 Er-2 strain 317/402 (Narine, Vitamax-E)*.

*+, C. albicans-carriers*.

*−, C. albicans non-carriers*.

*P_1_—comparison of male/female FMF patients within the investigated group*.

*P_2_—comparison with the control FMF patients*.

*P_3_—comparison with the placebo group patients*.

*^b^No significant differences in *C. albicans* presence (both yeast numbers and patients’ number) was observed in the placebo-treated group*.

*^c^Probiotic treatment lowered the C. albicans titer in all C. albicans-carriers*.

Fecal material was collected twice, before prescribing the probiotic and placebo and immediately after discontinuation of the treatment.

The age range of participants was 18–50 years. All patients’ diagnoses were confirmed by genetic analysis. None of the study participants had been treated with antibiotics, probiotics, hormones, or chemotherapeutic agents during the month leading up to the study. The duration of the colchicine treatment by patients was more than 7 year. During the 30-day period of study, patients with FMF used their regular colchicine medication (1 mg daily).

Study participants collected the fecal materials themselves in sterile plastic bags and transferred them to the laboratory not later than 2 h after collection.

The ZR Fecal DNA MiniPrep (Zymo Researc, Irvine, CA, USA) and the UltraClean^®^ Tissue & Cells DNA Isolation Kit (QIAGEN, Germantown, MD, USA) were used for total genomic DNA isolation following the manufacturers’ recommendations.

The primer sequences used for microarrays and 16S rRNA clone libraries were: 27f.jgi (Bacteria-specific) 5′-AGAGTTTGATCCTGGCTCAG-3′ and 1492r.jgi (Bacteria/Archaea-specific) 5′-GGTTACCTTGTTACGACTT-3′.

The fecal bacterial communities were assessed by a third generation, culture-independent, high-density DNA microaray (PhyloChip™; Affymetrix, Santa Clara, CA, USA) analysis as previously described (25). This approach detects and measures the relative abundance of more than 50,000 individual microbial taxa. This approach is based on the analysis of the sequence of 16S ribosomal RNA genes. The PhyloChip™ relies on the analysis of all nine variable regions of the 16S gene and offers deeper taxonomic classification than other general approaches. With 1.2 million probes per chip, the microchip-based hybridization approach ensures that measurements on key low-abundance bacteria are not suppressed by dominant members of the microbial community. Nearly full-length 16S rRNA-gene fragments were amplified using universal bacterial primers. The amplicons were used for PhyloChip™ analysis, assessing the differences in hybridization intensity—reflective of differences in the relative abundance of bacterial taxa (25).

*Candida albicans* in fecal samples was quantified on Brilliance™ Candida agar (Thermo Scientific, Waltham, MA, USA) and confirmed by a *C. albicans* PCR kit (DNA-Technology LLC, Russia).

Statistical analyses were performed by the Multibase 2015 Excel Add-in program (NumericalDynamics, Tokyo, Japan).

## Results

### *C. albicans* In FMF Patients

Despite the prevalence of men among Armenian FMF patients (16, 26) and the indication of possible associations between *C. albicans* infections and gender (27), our investigations showed that the number of *C. albicans*-carrier male FMF patients did not differ from the number of female carriers. Eleven out of 24 male FMF volunteers and 11 out of 24 female FMF volunteers had *C. albicans* counts above baseline levels in the gut microbiota before the probiotic treatment.

#### Effects of Probiotic on Gut *C. albicans* in FMF Patients

Probiotic treatment lowered the *C. albicans* titer in the FMF patient community. Only one “non-trial” female FMF patient with 10^7^ CFU/g *C. albicans* in fecal material and one “non-trial” male patient with 10^6^ CFU/g *C. albicans* in fecal material carried *C. albicans* at levels 10^4^ CFU/g fecal materials after probiotic treatment.

As expected, no significant differences in *C. albicans* presence (both yeast numbers and patients’ number) was observed in the placebo-treated group.

### Culture-Independent Analysis of *Enterobacteriaceae* in the Gut Microbiota of FMF Patients

**Figure 1:**
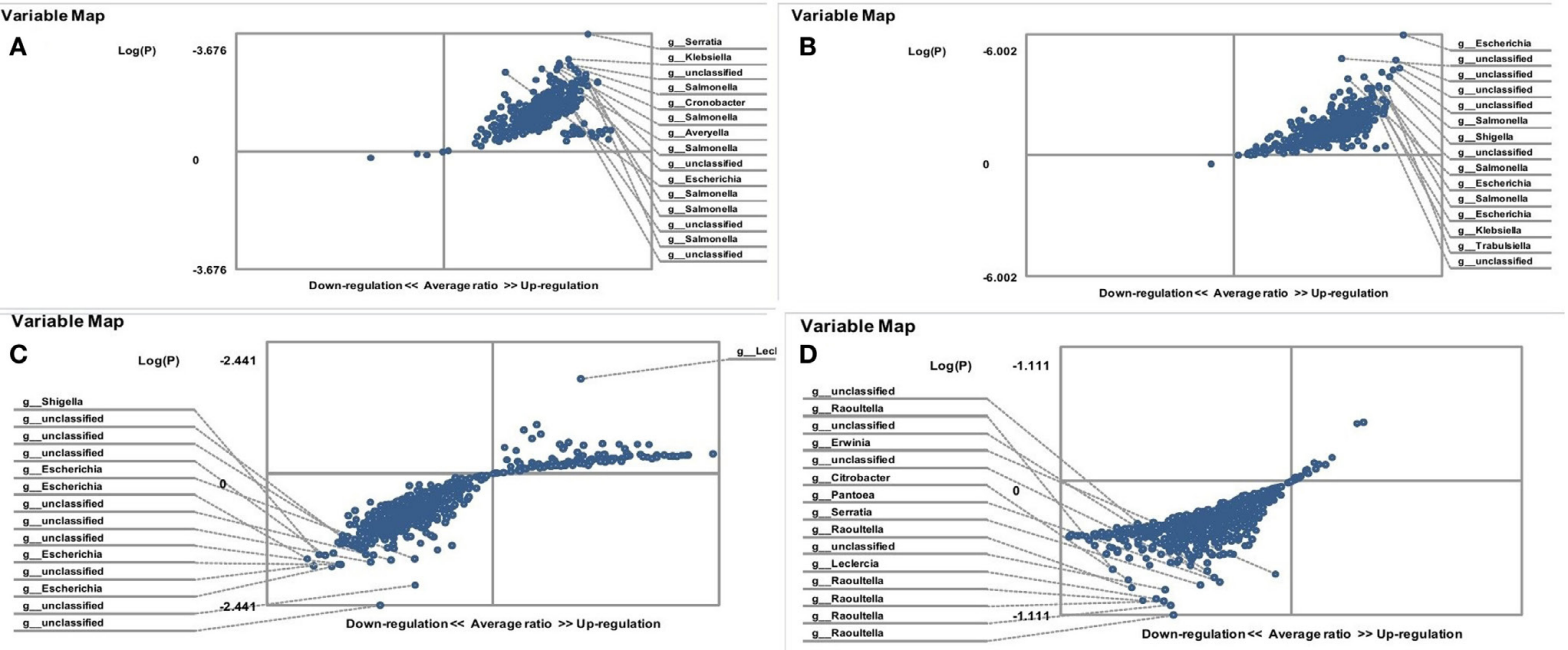
Distribution of gut *Enterobacteriaceae* in *Candida albicans*-carrier familial Mediterranean fever (FMF) patients. Contribution of variables to differentiation between two groups [*X* axis, average ratio between two groups; *Y* axis, −Log (P)]. The upper-right and lower-left corner are the most significant. **(A)** FMF women with gut *C. albicans* numbers above baseline level/FMF men with gut *C. albicans* numbers above baseline level; **(B)** FMF women with gut *C. albicans* numbers above baseline level before probiotic therapy/Healthy women; **(C)** FMF women with gut *C. albicans* numbers above baseline level after probiotic therapy/FMF women with gut *C. albicans* numbers above baseline level before probiotic therapy; **(D)** FMF men with gut *C. albicans* numbers above baseline level/Healthy men.

The relative abundance of enterobacteria in the gut microbiota of *C. albicans*-carrier patients is presented in Figure 1. The results show that female *C. albicans*-carrier FMF patients differ by their gut microbiota composition from that of male patients. Compared with diseased men, these women carried higher numbers of operational taxonomic units (OTUs) of the genera *Salmonella, Escherichia, Averyella, Cronobacter, Klebsiella*, and *Serratia* (*P* < 0.00021) (Figure 1A). Differences between the hybridization scores of male/female FMF patients show that the average intensity value of bacteria belonging to *Enterobacteriaceae* spp. is higher in the gut microbiota of *C. albicans*-carrier FMF women than in FMF men (8,141 ± 473 vs. 6,417 ± 347; *P* < 0.05) (Table 1). There was no detectable significant difference between OTUs in *C. albicans*-carrier male patients as compared to healthy people (*P* > 0.05) (Figure 1D).

The comparison of OTUs of enteric bacteria in *C. albicans*-carrier and non-carrier female patients revealed a statistically significant increase in OTUs of *Klebsiella* and *Erwinia* spp. in *C. albicans*-carriers (*P* = 0.011). On the other hand, the diversity of enterobacteria of *C. albicans*-carrier female patients significantly differed from that of healthy people (Figure 1B). Compared with the healthy women, *C. albicans*-carriers contain a high OTUs for the genera *Escherichia, Salmonella, Shigella, Klebsiella, and Trabulsiella* (*P* < 10^−5^).

#### Effects of Probiotic on Gut *Enterobacteriaceae*

Treatment with probiotic Narine led to a decrease in OTUs of the fecal *Enterobacteriaceae*, especially in the genera *Escherichia* and *Shigella*, in *C. albicans*-carrier FMF women (*P* = 0.0036) (Figure 1C).

The placebo led to an increase in hybridization scores for gut *Enterobacteriaceae*, both in male [6,992 ± 558 (*C. albicans* non-carriers, placebo group) vs. 5,208 ± 459 (*C. albicans* non-carriers, control group); *P* < 0.05 and 5,267 ± 325 (*C. albicans*-carriers, placebo group) vs. 4,853 ± 378 (*C. albicans*-carriers, control group); *P* > 0.05] and female [9,590 ± 794 (*C. albicans none*-carriers, placebo group) vs. 6,417 ± 347 (*C. albicans none*-carriers, control group); and 9,780 ± 422 (*C. albicans*-carriers, placebo group) vs. 8,141 ± 473 (*C. albicans*-carriers, control group); *P* < 0.05] FMF patients, while the probiotic Narine decreased these scores for gut *Enterobacteriaceae* in FMF patients, overall (Table 1).

### Culture-Independent Analysis of Lactobacilli in the Gut Microbiota of FMF Patients

**Figure 2:**
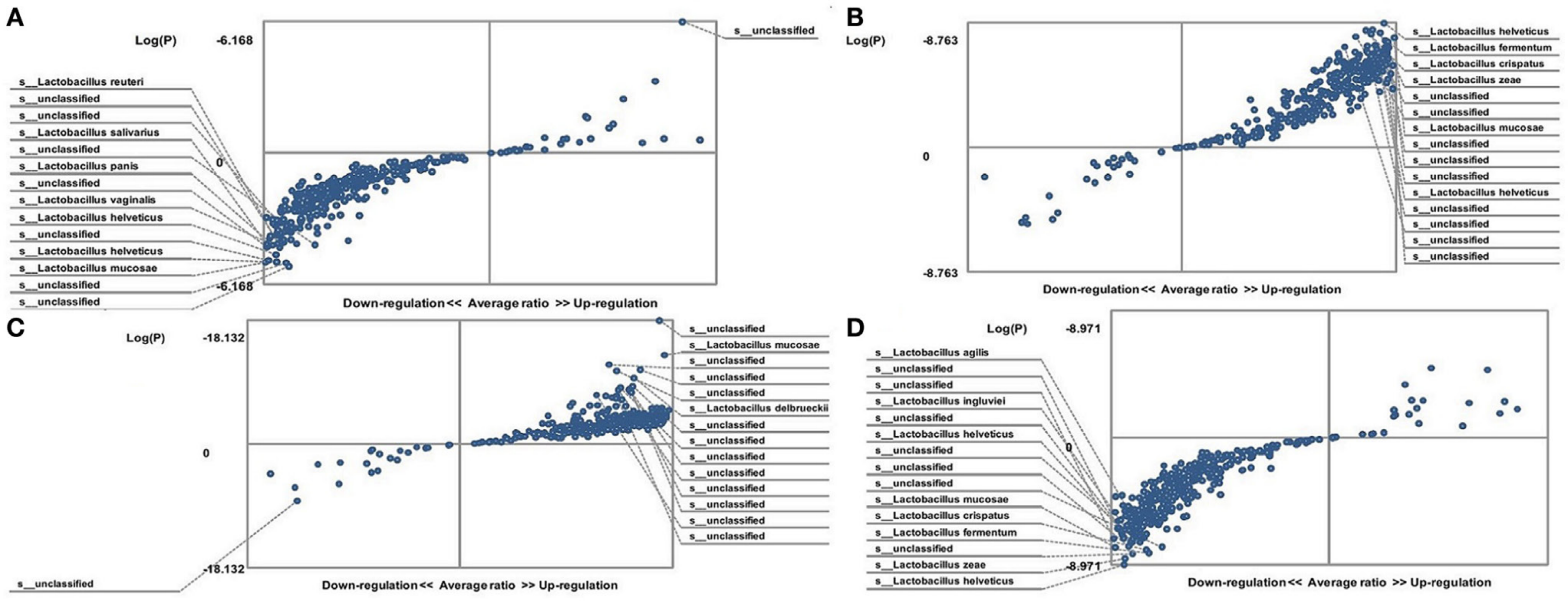
Distribution of gut lactobacilli in *Candida albicans*-carrier familial Mediterranean fever (FMF) patients. Contribution of variables to differentiation between two groups [*X* axis, average ratio between two groups; *Y* axis, −Log(P)]. The upper-right and left-down corner are the most significant. **(A)** FMF women with gut *C. albicans* numbers above baseline level/FMF women with *C. albicans* below baseline level; **(B)** FMF men with *C. albicans* below baseline level/healthy men; **(C)** FMF women with *C. albicans* below baseline level/healthy women; **(D)** FMF men with gut *C. albicans* numbers above baseline level/FMF men with *C. albicans* below baseline level.

A statistically significant decrease in lactobacilli was detected in the gut microbiota of FMF *C. albicans*-carriers (3,192 ± 107 vs. 3,556 ± 97; *P* < 0.05 and 2,834 ± 112 vs. 4,112 ± 93) (Table 1). The gut microbiota of FMF patients (both male and female) with *C. albicans* numbers below baseline levels contained a high OTU numbers of lactobacilli when compared with *C. albicans*-carriers (*P* < 10^−5^) (Figures 2A,D). Both male and female *C. albicans*-carrier FMF patients were closer to the healthy volunteers in the relative abundance of lactobacilli than to FMF patients overall. FMF patients with *C. albicans* below baseline levels showed a higher abundance of lactobacilli compared to healthy volunteers and *C. albicans*-carriers (*P* < 10^−5^) (Figures 2C,B).

We detected some changes in hybridization scores for a diversity of lactobacilli in FMF patients after taking placebo (4,560 ± 299 vs. 3,556 ± 97, 4,360 ± 173 vs. 3,192 ± 107, 4,978 ± 138 vs. 4,112 ± 93, and 3,813 ± 201 vs. 2,834 ± 112; *P* < 0.05). Probiotic uptake had no statistically significant effect on the gut lactobacilli of these patients (4,793 ± 289 vs. 4,560 ± 299, 3,834 ± 263 vs. 4,360 ± 273, 4,873 ± 185 vs. 4,978 ± 138, and 3,941 ± 217 vs. 3,813 ± 201; *P* > 0.05) (Table 1).

### Culture-Independent Analysis of *Staphylococcus aureus* in the Gut Microbiota of FMF Patients

Changes were detected in *S. aureus* abundance between *C. albicans*-carrier and non-carrier FMF women. The female FMF cohort with *C. albicans* numbers above baseline levels had a low abundance of *S. aureus* (data not shown). There were no statistically significant changes in the abundance of *S. aureus* between *C. albicans*-carrier and non-carrier FMF men and between FMF and healthy men (*P* > 0.05).

#### Effects of Probiotic on Gut *S. aureus*

The investigation also explored if there were any differences in the abundance of gut *S. aureus* in FMF women before and after probiotic administrations. The results showed that the probiotic had no significant effect on *S. aureus* levels in FMF women (*P* > 0.05) (data not shown).

### Culture-Independent Analysis of *Enterococcus faecalis* in the Gut Microbiota of FMF Patients

**Figure 3:**
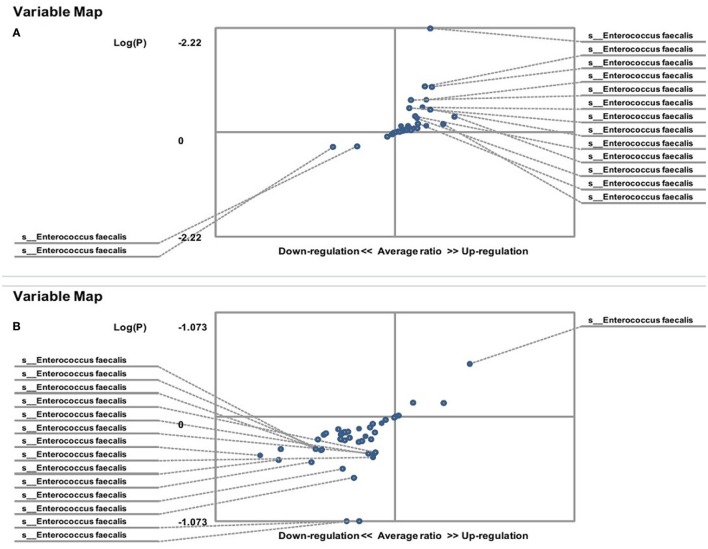
Distribution of gut *Enterococcus faecalis* in *Candida albicans-*carrier familial Mediterranean fever (FMF) patients. Contribution of variables to differentiation between two groups [*X* axis, average ratio between two groups; *Y* axis, −Log (P)]. The upper-right and left-down corner are the most significant. **(A)** FMF men with gut *C. albicans* numbers above baseline level/FMF men with *C. albicans* below baseline level; **(B)** FMF women with gut *C. albicans* numbers above baseline level/FMF women with *C. albicans* below baseline level.

The hybridization scores for *E. faecalis* indicate differences between both male and female FMF *C. albicans*-carriers compared with non-carriers (*P* < 0.05) (Table 1). Despite this, these data show no significant differences in the relative abundance of *E. faecalis* between female FMF *C. albicans*-carriers compared with non-carriers (*P* > 0.08), while male FMF *C. albicans*-carriers have increased abundance of *E. faecalis* (*P* = 0.006) in their gut microbiota compared with that of male patients with *C. albicans* below baseline levels (Figures 3A,B).

**Figure 4:**
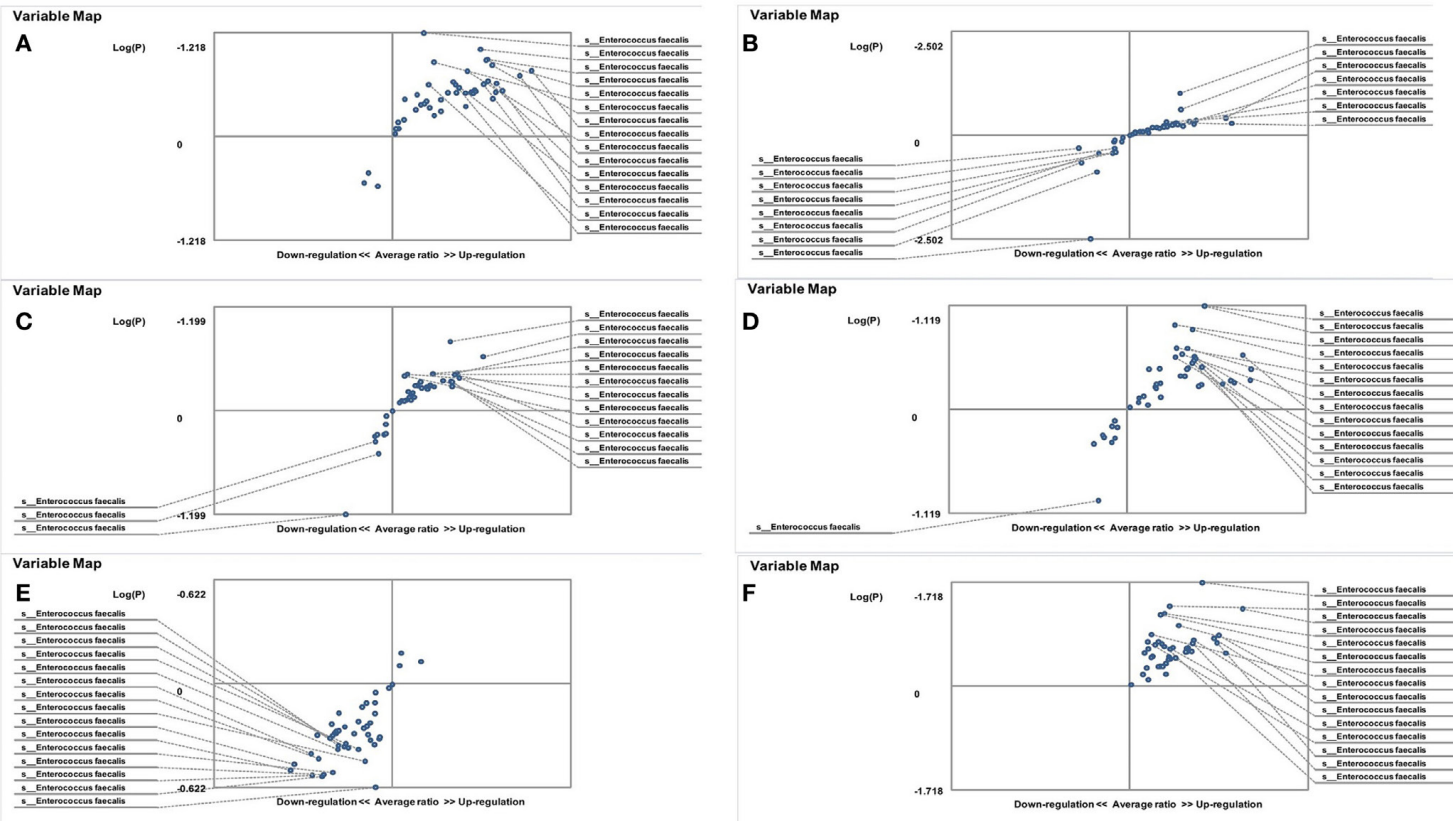
Distribution of gut *Enterococcus faecalis* in familial Mediterranean fever (FMF) carrier women. Contribution of variables to differentiation between two groups [*X* axis, average ratio between two groups; *Y* axis, −Log (P)]. The upper-right and lower-left corner are the most significant. **(A)** FMF women with *Candida albicans* below baseline level/healthy women; **(B)** FMF women with gut *C. albicans* numbers above baseline level/healthy women; **(C)** FMF women with *C. albicans* below baseline level after probiotic therapy/healthy women; **(D)** FMF women with gut *C. albicans* numbers above baseline level after probiotic therapy/healthy women; **(E)** FMF women with *C. albicans* below baseline level after probiotic therapy/FMF women with *C. albicans* below baseline level before probiotic therapy; **(F)** FMF women with gut *C. albicans* numbers above baseline level after probiotic therapy/FMF women with gut *C. albicans* numbers above baseline level before probiotic therapy.

**Figure 5:**
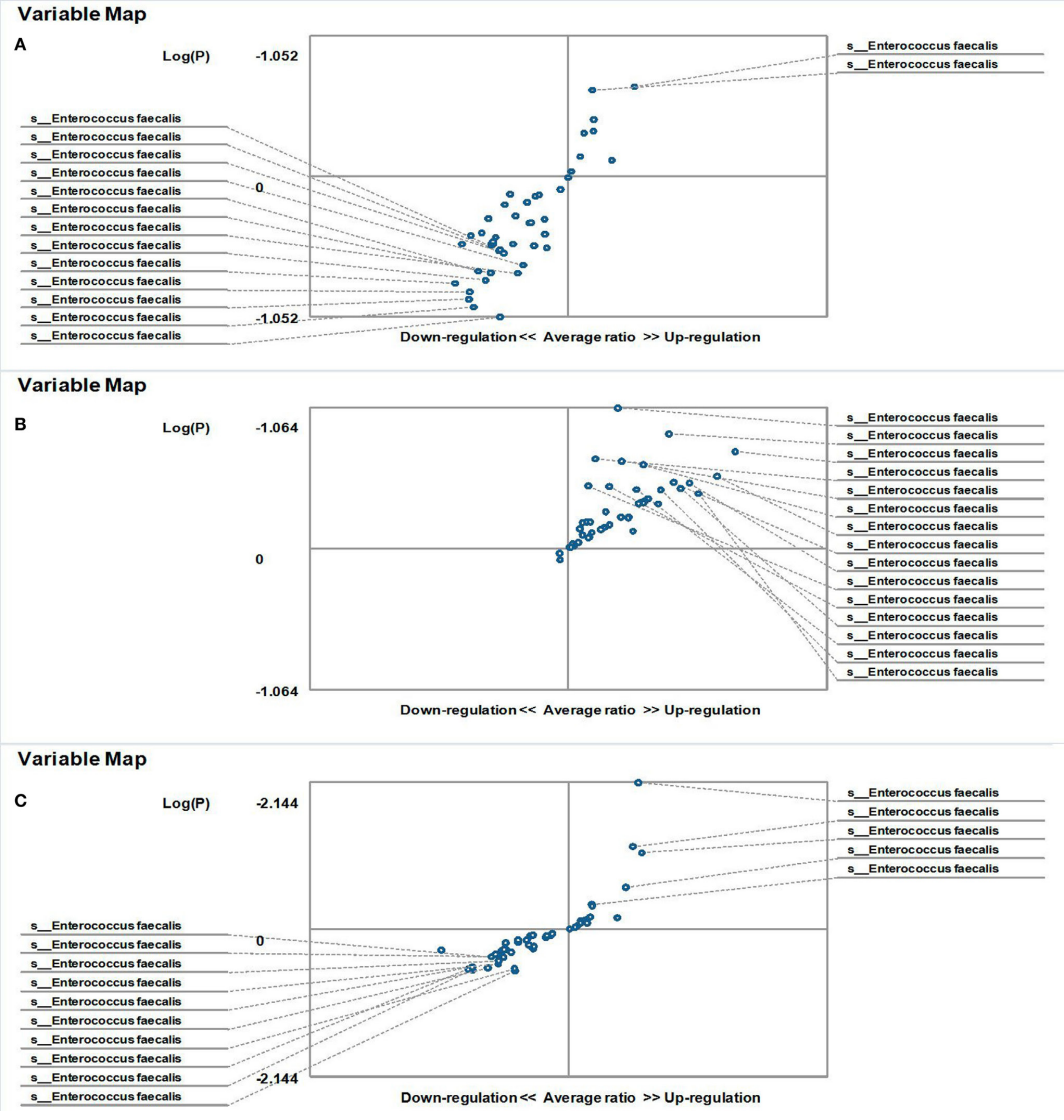

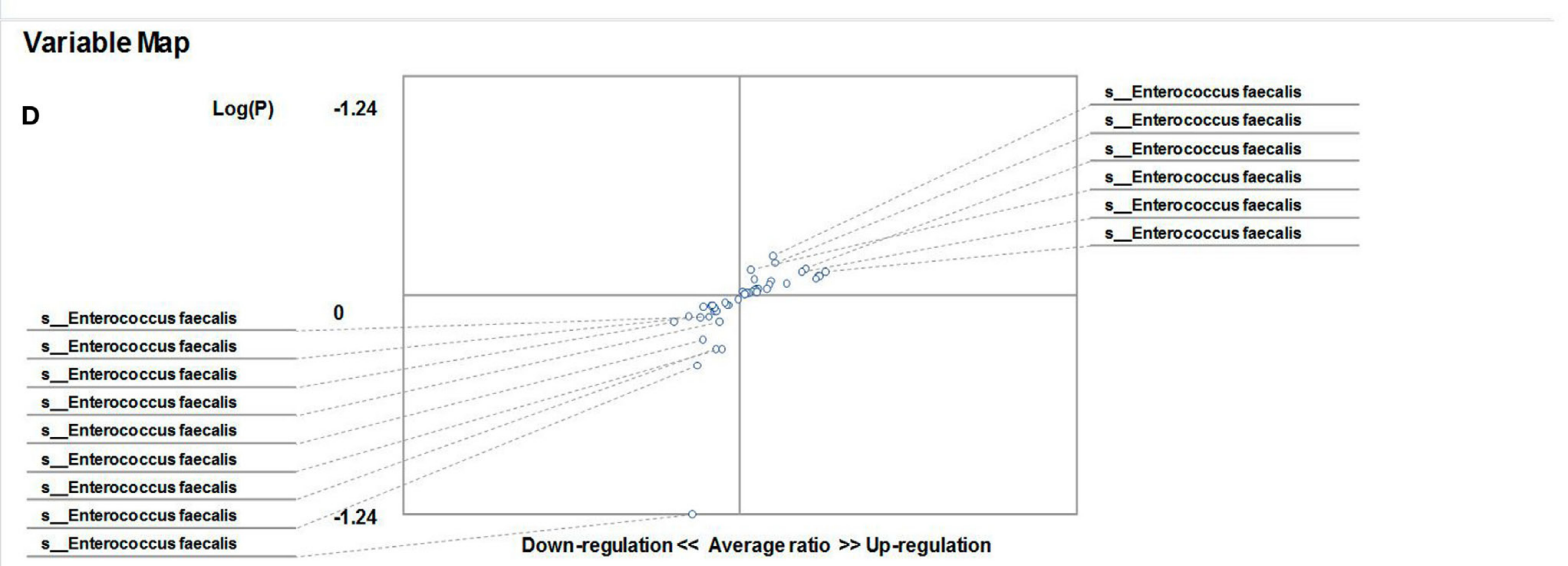
Distribution of gut *Enterococcus faecalis* in familial Mediterranean fever (FMF) men. Contribution of variables to differentiation between two groups [*X* axis, average ratio between two groups; *Y* axis, −Log (P)]. The upper-right and lower-left corner are the most significant. **(A)** FMF men with *Candida albicans* below baseline level/healthy men; **(B)** FMF men with gut *C. albicans* numbers above baseline level after probiotic therapy/healthy men; **(C)** FMF men with gut *C. albicans* numbers above baseline level/Healthy men; **(D)** FMF men with gut *C. albicans* numbers above baseline level before probiotic therapy/FMF men with gut *C. albicans* numbers above baseline level after probiotic therapy.

On the other hand, the results showed that both groups of FMF women, *C. albicans*-carriers and non-carriers, differ from healthy volunteers in the abundance of *E. faecalis* in the gut microbiota (*P* < 0.05) (Figures 4A,B). There were no differences between healthy men and FMF men in terms of *E. faecalis* abundance in their gut microbiota (*P* > 0.05) (Figure 5A), while *C. albicans*-carrier FMF men differed from healthy men volunteers (*P* < 0.05) (Figure 5C).

#### Effects of Probiotic Administration on Gut *Enterococcus faecalis*

Probiotic-therapy reduced the number of yeast in *C. albicans*-carrier women. However, there were no statistically significant differences between *C. albicans*-carrier/healthy women and *C. albicans*-non-carrier/healthy women in their gut *E. faecalis* abundances (*P* > 0.05) (Figures 4C,D) as well as differences between *C. albicans*- none carrier women before and after the probiotic administration (*P* > 0.05) (Figure 4E), in spite of this, there were statistically significant differences between *C. albicans*-carrier and *C. albicans*-non-carrier women in their gut *E. faecalis* abundances (*P* < 0.05) (Figure 4F). The placebo groups showed increase in hybridization scores of gut *E. faecalis* both in male (6,374 ± 169 vs. 4,441 ± 128; *P* < 0.05 and 5,689 ± 109 vs. 4,928 ± 144; *P* < 0.05) and female (6,534 ± 205 vs. 6,405 ± 157; and 5,070 ± 244 vs. 4,168 ± 213; *P* < 0.05) FMF patients. Compared to the placebo effect, the statistically significant increase in scores of gut *E. faecalis* was attributed to male FMF patients not carrying *C. albicans* treated with the probiotic (7,108 ± 273 vs. 5,689 ± 109; *P* < 0.05), while the probiotic-therapy decreased *E. faecalis* scores in *C. albicans*-carrier and non-carrier female patients (3,771 ± 123 vs. 5,070 ± 244 and 5,602 ± 189 vs. 6,534 ± 205; *P* < 0.05) (Table 1). After the treatment with commercial probiotic Narine, there were no statistically significant differences between healthy men and FMF men with *C. albicans*-carriers in gut *E. faecalis* (Figure 5B). Statistically significant differences between FMF men with *C. albicans*-carriers before and after the treatment with commercial probiotic Narine were observed (Figure 5D).

## Discussion

The differences in the pathogenesis of infectious diseases in male and female patients were previously reported (28), and the significant influences of gender on the development of autoimmune disease have been described (28, 29). It was reported that a female steroid hormone, estrogen, probably playing a role in gut tight junction expression and permeability, might also be the reason for female autoimmune diseases (30). Moreover, the interaction between gender-specific immune differences and the specific immune response to individual microbes was reported previously (31). There are some indications of a possible association between *C. albicans* infections and gender in the literature as well (32, 33).

According to our investigations, the patients’ gender is one of the main factors affecting the composition of gut microbiota in FMF patients despite the lack of previous report on this difference. The male/female ratio of Armenian FMF patients with MEFV mutation patterns M694V/V726A, according to reported data, is 1.16–1.2:1 (16, 26, 34). In addition, our previous studies revealed gender differences in several blood parameters of FMF patients, such as ESR and CRP (35).

Gender-based differences in the relationship of IL-6 signaling and adrenocorticotropic hormone, which is usually produced in response to biological stress, was reported previously (36), and the relationships between triggering factors and FMF were reported for Armenian patients (37). The experiments included 177 male and 98 female FMF patients and showed that emotional stress was one of the most common triggering factors for FMF attacks with serositis (49.8%) after cold exposure (59.3%) (38). Moreover, the relationships between triggering factors and both M694V (with starvation) and V726A (with long-duration travel) alleles were revealed (38).

An important role for IL-6 in the immune response to *E. coli* and *C. albicans* was reported earlier using *Il6* null mice (*Il6^−/−^*) (39, 40). Thus, Il-6 with its pro- and anti-inflammatory characteristics being responsible for the transition of innate-acquired immunity, could participate in “immune dimorphism” in male and female FMF patients. Generally, there should be differences between the specific bacterial OTUs (*Enterobacteriaceae*/lactobacilli/*E. faecalis*/*S. aureus*) of healthy people and *C. albicans*-carrier FMF patients, as well as in *C. albicans*-carrier and non-carrier FMF patients. These differences should be expected if the relationship between the investigated gut bacteria and *C. albicans* are critical for the yeast’s increased number in the gut microbiota of FMF patients.

According to our investigations, the diversity of enterobacteria in female *C. albicans*-carrier patients significantly differs from those in healthy participants and from those in female non-carrier patients. In addition, female *C. albicans* non-carrier patients were having a wider diversity of *Enterobacteriaceae* compared with the healthy women. We did not observe differences between the *Enterobacteriaceae* OTUs of healthy men and *C. albicans*-carrier FMF men despite the fact that probiotic treatment decreased the relative abundance of gut *Enterobacteriaceae* in *C. albicans*-carrier FMF men, too.

Possibly, the high diversity of *Enterobacteriaceae* in the gut microbiota of FMF patients may have an effect on the overgrowth of *C. albicans* in FMF women. The tendency of the simultaneous “normalization” of OTUs for enteric bacteria and C*. albicans* in the gut microbiota of female FMF patients after the probiotic Narine treatment supports this hypothesis. However, there was no such tendency detected in FMF male subjects.

Based on the effects of probiotic on the relative abundance of fecal-*Enterobacteriaceae*, we suggest that mechanisms of probiotic effects on gut *C. albicans* are different for male and female FMF patients. Similar analysis revealed a potential association between the OTUs of *E. faecalis* and *C. albicans* in the gut microbiota of male FMF patients. In contrast to fecal *Enterobacteriaceae* and *E. faecalis* OTUs, the gut microbiota of FMF patients (both male and female) with *C. albicans* below baseline levels contains a relatively high abundance of lactobacilli compared with *C. albicans*-carriers. However, the probiotic supplementation did not affect the abundance of lactobacilli in *C. albicans*-carrier FMF women and men. The primary comparative analysis on *S. aureus* OTUs in *C. albicans*-carrier/non-carrier FMF and healthy people does not support the hypothesis of potential strong synergistic interaction between *S. aureus* and *C. albicans*.

Although the probiotic *L. acidophilus INMIA 9602 Er* strain *317/402* does not have any effect on *C. albicans in vitro* (16), the number of FMF patients in remission (both male and female) who carried *C. albicans* above baseline levels, decreased after probiotic therapy in the double blind, partly randomized, placebo-controlled trial on 48 volunteer patients with FMF in remission. The abovementioned data show that greater relative abundances of enteric bacteria, especially *Klebsiella*, in *C. albicans*-carrier female FMF patients and *E. faecalis* in *C. albicans*-carrier male FMF patients may contribute to the increase in numbers of *C. albicans* in FMF patients. The report on antagonistic activity of the probiotic against *Klebsiella in vitro* supports this hypothesis (16).

Different inflammasomes are shown to be involved in the response to a *C. albicans* infection, including the NLRP_3_ inflammasome with a caspase recruitment domain and caspase-1, and the production of interleukin-1 beta (IL-1β) and IL-18 through the NLRP_3_ inflammasome is crucial for adaptive cellular protection against *C. albicans* (41). Another inflammasome, NLRC_4_, involved in the response to *C. albicans* infection (42), is able to recognize flagellin and components of the bacterial secretion systems providing host defense to a range of pathogens including *K. pneumoniae* (43) and *Yersinia* (44). NLRP_3_ and NLRC_4_ together allow for the recognition of different danger signals from the same pathogenic infection (45). Wild-type pyrin negatively modulates NLRP_3_ inflammasome-dependent IL-1β release (46).

Our investigations showed that the pyrin inflammasome mutation M694V/V726A, leading to FMF disease, effects the gut microbiota and contributes to differences between male and female patients. The number of *C. albicans*-carrier male FMF patients did not differ from the number of female carriers, indicating that there was no direct association between the host’s genetics and *C. albicans*-carriage. However, the considerable effects of other factors, such as comparatively high concentrations and/or long-term colchicine use (with other environmental factors) may outweigh the effects of the host’s genetic background.

The most effective long-term treatment for FMF is the administration of colchicine (47), which inhibits inflammasome activation within macrophages (48, 49). There is no indication of a different effect of the treatment between male and female patients. Most likely, colchicine inhibits NLRP_3_ inflammasome-dependent IL-1β release, thereby influencing the overgrowth of *C. albicans* in the gut microbiota of FMF patients.

Our investigations indicate that the uptake of *L. acidophilus* INMIA 9,602 Er 317/402 reduces the numbers of yeast in the gut microbiota of FMF patients.

Future microbiological and immunological studies will be aimed at clarification of the functional aspects and the detailed mechanisms of action of *Lactobacillus acidophilus* INMIA 9,602 Er 317/402 and its impact on human health in relation to FMF. This will assist in designing “individual diets” for FMF patients.

## Ethics Statement

This study was performed in accordance with institutional ethical guidelines and was approved by the Ethics Committee at the Ministry of Education and Science of Armenia. All investigated patients gave written informed consent prior to the study.

## Author Contributions

All authors have made a substantial, direct, and intellectual contribution to the work and have approved it for publication. AP and TT conceived and designed the experiments. MB, AM, SP, SPet, LG, and VT performed the experiments. AP analyzed the data and wrote the paper. TT, MC, and SK reviewed data analysis and the manuscript. All authors reviewed the manuscript.

## Conflict of Interest Statement

The authors declare that the research was conducted in the absence of any commercial or financial relationships that could be construed as a potential conflict of interest.
